# Improving protein secondary structure prediction based on short subsequences with local structure similarity

**DOI:** 10.1186/1471-2164-11-S4-S4

**Published:** 2010-12-02

**Authors:** Hsin-Nan Lin, Ting-Yi Sung, Shinn-Ying Ho, Wen-Lian Hsu

**Affiliations:** 1 Bioinformatics Program, Taiwan International Graduate Program, Academia Sinica, Taipei, Taiwan; 2 Bioinformatics Lab., Institute of Information Science, Academia Sinica, Taipei, Taiwan; 3 Institute of Bioinformatics, National Chiao Tung University, Hsinchu, Taiwan

## Abstract

**Background:**

When characterizing the structural topology of proteins, protein secondary structure (PSS) plays an important role in analyzing and modeling protein structures because it represents the local conformation of amino acids into regular structures. Although PSS prediction has been studied for decades, the prediction accuracy reaches a bottleneck at around 80%, and further improvement is very difficult.

**Results:**

In this paper, we present an improved dictionary-based PSS prediction method called SymPred, and a meta-predictor called SymPsiPred. We adopt the concept behind natural language processing techniques and propose synonymous words to capture local sequence similarities in a group of similar proteins. A synonymous word is an *n-*gram pattern of amino acids that reflects the sequence variation in a protein’s evolution. We generate a protein-dependent synonymous dictionary from a set of protein sequences for PSS prediction.

On a large non-redundant dataset of 8,297 protein chains (*DsspNr-25*), the average *Q*_3_ of SymPred and SymPsiPred are 81.0% and 83.9% respectively. On the two latest independent test sets (*EVA Set_1* and *EVA_Set2*), the average *Q*_3_ of SymPred is 78.8% and 79.2% respectively. SymPred outperforms other existing methods by 1.4% to 5.4%. We study two factors that may affect the performance of SymPred and find that it is very sensitive to the number of proteins of both known and unknown structures. This finding implies that SymPred and SymPsiPred have the potential to achieve higher accuracy as the number of protein sequences in the NCBInr and PDB databases increases.

**Conclusions:**

Our experiment results show that local similarities in protein sequences typically exhibit conserved structures, which can be used to improve the accuracy of secondary structure prediction. For the application of synonymous words, we demonstrate an example of a sequence alignment which is generated by the distribution of shared synonymous words of a pair of protein sequences. We can align the two sequences nearly perfectly which are very dissimilar at the sequence level but very similar at the structural level. The SymPred and SymPsiPred prediction servers are available at http://bio-cluster.iis.sinica.edu.tw/SymPred/.

## Background

Proteins can perform various functions when they fold into proper three-dimensional structures. However, since determining the structure of a protein through wet-lab experiments can be time-consuming and labor-intensive, computational approaches are preferable. To characterize the structural topology of proteins, Linderstrøm-Lang proposed the concept of a protein structure hierarchy with four levels: primary, secondary, tertiary, and quaternary. In the hierarchy, protein secondary structure (PSS) plays an important role in analyzing and modeling protein structures because it represents the local conformation of amino acids into regular structures. There are three basic secondary structure elements (SSEs): α-helices (H), β-strands (E), and coils (C). Many researchers employ PSS as a feature to predict the tertiary structure [1-4], function [5-8], or subcellular localization [9-11] of proteins. It is noteworthy that, among the various features used to predict protein function, such as amino acid composition, disorder patterns, and signal peptides, PSS makes the largest contribution [12]. Moreover it has been suggested that secondary structure alone may be sufficient for accurate prediction of a protein’s tertiary structure [13].

Current PSS prediction methods can be classified into two categories: template-based methods and sequence profile-based methods [14]. Template-based methods use protein sequences of known secondary structures as templates, and predict PSS by finding alignments between a query sequence and sequences in the template pool. The nearest-neighbor method belongs to this category. It uses a database of proteins with known structures to predict the structure of a query protein by finding nearest neighbors in the database. By contrast, sequence profile-based methods (or machine learning methods) generate learning models to classify sequence profiles into different patterns. In this category, Artificial Neural Networks (ANNs), Support Vector Machines (SVMs) and Hidden Markov Models (HMMs) are the most widely used machine learning algorithms [15-21]. Template-based methods are highly accurate if there is a sequence similarity above a predefined threshold between the query and some of the templates; otherwise, sequence profile-based methods are more reliable. However, the latter may under-utilize the structural information in the training set when the query protein has some sequence similarity to a template in the training set [14]. An approach that combines the strengths of both types of methods is required for generating reliable predictions irrespective of whether the query sequence is similar or dissimilar to the templates in the training set.

To measure the accuracy of secondary structure prediction methods, researchers often use the average three-state prediction accuracy (*Q*_3_) accuracy or the segment overlap (SOV) measure [22,23]. The estimated theoretical limit of the accuracy of secondary structure assignment from the experimentally determined 3D structure is 88% of the *Q*_3_ accuracy [5,24], which is deemed the upper bound for secondary structure prediction. However, PSS prediction has been studied for decades and has reached a bottleneck, since the *Q*_3_ accuracy remains at approximately 80 % and further improvement is very difficult, as demonstrated by the CASP competitions. Currently, the most effective PSS prediction methods are based on machine learning algorithms, such as PSIPRED [17], SVMpsi [19], PHDpsi [25], Porter [26] and SPINE [27], which employ ANN or SVM learning models. The two most successful template-based methods are NNSSP [28,29] and PREDATOR [30]. They use the structural information obtained from local alignments among query proteins and template proteins, and their *Q*_3_ accuracy is approximately 70%. Thus, the difference in the accuracy of the two categories is approximately 10%.

In a previous work on PSS prediction [31], we proposed a method called PROSP, which utilizes a sequence-structure knowledge base to predict a query protein’s secondary structure. The knowledge base consists of sequence fragments, each of which is associated with a corresponding structure profile. The profile is a position specific scoring matrix that indicates the frequency of each SSE at each position. The average *Q*_3_ accuracy of PROSP is approximately 75%.

In this paper, we present an improved version of PROSP called SymPred, which is a dictionary-based method for predicting the secondary structure of a protein sequence. Dictionary-based approaches are widely used in the field of natural language processing (NLP). We generate synonymous words from a protein sequence and its similar sequences. The definition of a synonymous word is given in the Methods section. The major differences between SymPred and PROSP are as follows. First, the constitutions of the dictionary (SymPred) and the knowledge base (PROSP) are different. Second, the scoring systems of SymPred and PROSP are different. Third, unlike PROSP, SymPred allows inexact matching. Our experiment results show that SymPred can achieve 81.0% *Q*_3_ accuracy on a non-redundant dataset, which represents a 5.9% performance improvement over PROSP.

There are significant differences between SymPred and other methods in the two categories described earlier. First, in contrast to template-based methods, SymPred does not generate a sequence alignment between the query protein and the template proteins. Instead, it finds templates by using local sequence similarities and their possible variations. Second, SymPred is not a machine learning-based approach. Moreover, it does not use a sequence profile, so it cannot be classified into the second category. However, like machine learning-based approaches, SymPred can capture local sequence similarities and generate reliable predictions. Therefore, SymPred combines the strengths of template-based and sequence profile-based methods. The experiment results on the two latest independent test sets (*EVA_Set 1* and *EVA_Set2*) show that, in terms of *Q*_3_ accuracy, SymPred outperforms other existing methods by 1.4% to 5.4%.

The remainder of this paper is organized as follows. In the Methods section, we define synonymous words, and describe the method used to construct the protein-dependent synonymous dictionary. We also discuss the SymPred algorithm and the integrated SymPsiPred method. In the Results section, we compare the performance of SymPred and SymPsiPred with that of other methods. We also examine two factors that may affect SymPred’s performance. In the Discussion section, we analyze the prediction power of SymPred on similar proteins as well as the relationship between the number of synonymous words and the method’s prediction performance. We also demonstrate an example of a sequence alignment generated by the distribution of shared synonymous words of a pair of protein sequences. We can align the two sequences nearly perfectly which are very dissimilar at the sequence level but very similar at the structural level.

## Methods

### Synonymous words versus similar words

It is well known that a protein structure is encoded and determined by its amino acid sequence. Therefore, a protein sequence can be treated as a text written in an unknown language whose alphabet comprises 20 distinct letters; and the protein’s structure is analogous to the semantic meaning of the text. Currently, we cannot decipher the “protein language” with existing biological experiments or natural language processing (NLP) techniques; thus, the translation from sequence to structure remains a mystery. However, biologists have found that two proteins with a sequence identity above 40% may have a similar structure and function. The high degree of robustness of the structure with respect to the sequence variation shows that the structure is more conserved than the sequence.

**Figure 1:**
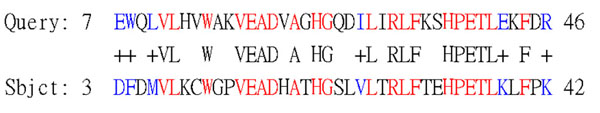
**A local sequence alignment derived by PSI-BLAST.** The identical residues are labelled with letters and conserved substitutions are labelled with + symbols. The alignment in this example shows that the sequence fragment from position 7 to position 46 of the query sequence is very similar to that from position 3 to position 42 in the subject sequence. It is assumed that the two sequences have a similar semantic relation because they form a significant sequence alignment.

In evolutionary biology, protein sequences that derive from a common ancestor can be traced on the basis of sequence similarity. Such sequences are referred to as homologous proteins. In terms of natural language, a group of homologous protein sequences can be treated as texts whose semantic meaning is identical or similar. The homologous relationship between proteins can be always captured by sequence alignment; thus, we assume that two sequence fragments have a similar semantic relation if they can be aligned by a sequence alignment tool, such as BLAST, with a significant e-value, say 0.001. Figure 1 shows an example of a sequence alignment derived by BLAST with an e-value of 0.001. In the alignment, the identical residues are labelled with letters and conserved substitutions are labelled with + symbols. The sequence identity between the two sequence fragments in this example is 50% (=20/40).

The idea of treating n-gram patterns as words has been widely used in biological sequence comparison methods; BLAST is probably the most well known method. BLAST’s heuristic algorithm uses a sliding window to generate an initial word list from a query sequence. To further expand the word list, BLAST defines a *similar word* with respect to a word on the list based on the score of the aligned word pair. A word whose alignment score is well above a threshold is called a similar word and is added to the list to recover the sensitivity lost by only matching identical words. However, in BLAST, the length of a word is only 2 or 3 characters (the default size) for protein sequences and short words are very likely to generate a large number of false hits of protein sequences that are not actually semantically related.

In this study, we define synonymous words as follows. Given a protein sequence *p,* we use PSI-BLAST to generate a number of significant sequence alignments between *p* and its similar proteins *sp.* All words, i.e., *n*-grams, in *p* and *sp* are generated by a sliding window of size *n.* Given a word *w* in *p,* the *synonymous word of w* is defined as the word *sw* in *sp* that is aligned with *w.* Please note that no gap is allowed in either *w* or *sw* since there is no structural information in the gap region. Thus, the major difference between synonymous words and similar words is that synonymous words are based on sequence alignments (i.e., they are context-sensitive), whereas similar words are based on word alignments (i.e., they are context-free). Take the sequence alignment in Figure 1 as an example. The *Sbjct* sequence is a similar protein to the *Query* sequence; therefore, DFDM is deemed synonymous to the word EWQL if the word length is 4, and FDMV is deemed synonymous to the next word WQLV. Based on the observation of the high robustness of structures, if the *Query* is of known structure and the *Sbjct* is of unknown structure, we assume that each synonymous word *sw* adopts the same structure as its corresponding word *w;* i.e., *sw* inherits the structure of *w.*

Moreover, different synonymous words *sw* for a word *w* should have different similarity scores to *w.* To estimate the similarity between *w* and *sw,* we calculate the *similarity level* according to the number of amino acid pairs that are *interchangeable.* If two amino acids are aligned in a sequence alignment, they are said to be *interchangeable* if they have a positive score in BLOSUM62. Since a protein word is an n-gram pattern, the range of the similarity level between the components of a word pair is from 0 to *n.* For example, in Figure 1, the similarity level between DFDM and EWQL is 3, and that between FDMV and WQLV is also 3.

### The advantages of synonymous words

The major advantages of using synonymous words over similar words are as follows. First, since the synonymous words are generated from a group of similar proteins, two irrelevant proteins will use different groups of similar proteins to generate their own synonymous words. Two irrelevant proteins are unlikely to have common synonymous words, even if their original sequences contain identical words. This observation implies that synonymous words are *protein-dependent.* Second, two remote homologous proteins are very likely to have common similar proteins because of the transitivity of the homology relationship, so they probably share some synonymous words. Third, a synonymous word is given a similarity score (i.e., the similarity level) respective to the word it is aligned with. Therefore, a synonymous word may have different similarity scores depending on which word it is aligned with. Accordingly, a synonymous word is a protein-dependent similar word that may also have a similar semantic meaning in terms of its structure.

In this study, we construct a protein-dependent synonymous word dictionary that lists possible synonyms for words of a protein sequence in a dataset. We use synonymous words as features to infer structural information for PSS prediction.

### Construction of a protein-dependent synonymous dictionary

Given a query sequence, we use PSI-BLAST to generate a number of significant alignments, from which we acquire possible sequence variations. In general, the similar protein sequences (i.e., the Sbjct sequences) reported by PSI-BLAST share highly similar sequence identities (between 25% and 100%) with the query, which implies that the sequences may have similar structures. Therefore, we identify synonymous words in those sequences.

Using a dataset of protein sequences with known secondary structures, we construct a protein-dependent synonymous dictionary, called *SynonymDict.* The dataset used to construct *SynonymDict* is described in the Results section. For each protein *p* in the dataset, we first extract protein words from its original sequence using a sliding window of size *n.* Each protein word, as well as the corresponding SSEs of the successive *n* residues, the protein source *p,* and the similarity level (here, the similarity level is *n*), are stored as an entry in *SynonymDict.* A protein source *p* represents the structural information provider. We then use PSI-BLAST to generate a number of similar protein sequences. Specifically, to find similar sequences, we perform a PSI-BLAST search of the NCBInr database with parameters *j*=3, *b*=500, and *e*=0.001 for each protein *p* in the dataset. Since the NCBInr database only contains protein sequence information, each synonymous word inherits the SSEs of its corresponding word in *p.* A PSI-BLAST search for a specific query protein *p* generates a number of local pairwise sequence alignments between *p* and its similar proteins. Statistically, an e-value of 0.001 generally produces a safe search and signifies sequence homology [32]. Similarly, each synonymous word and its inherited structure, the protein source *p,* and the similarity level are stored as an entry in *SynonymDict.*

**Figure 2:**
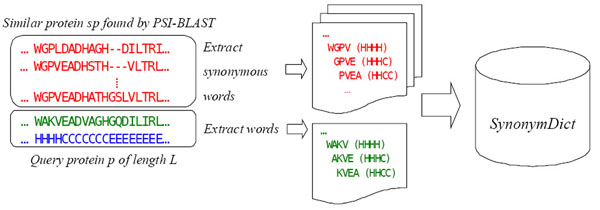
**The procedure used to extract protein words and synonymous words for a query protein *p*.** The procedure used to extract protein words and their synonymous words for a given query protein *p* (assuming the window size *n* is 4). We use a sliding window to screen the query sequence and all the similar protein sequences found by PSI-BLAST and extract all words. Each word is associated with a piece of structural information of the region from which it is extracted. The protein source of all the extracted words is the query protein *p*, since all the structural information is derived from *p*.

Figure 2 shows the procedure used to extract protein words and synonymous words for a query protein *p*. We use a sliding window to screen the query sequence, as well as all the similar protein sequences found by PSI-BLAST, and extract all words. The query protein *p* is the protein source of all the extracted words. Each word is associated with a piece of structural information of the region from which it is extracted. For example, WGPV is a synonymous word of WAKV. Since it is from a similar protein of unknown structure, it is associated with a piece of structural information of WAKV, which is HHHH.

Note that a synonymous word may appear in more than one similar protein when all similar protein sequences are screened. We cluster identical words together and store the frequency in the synonymous word entry. Table 1 shows an example of a synonymous word entry in *SynonymDict.* In the example, WGPV is a synonymous word of proteins *A, B* and C, since it is extracted from the similar proteins of *A, B* and *C.* The synonymous word inherits the corresponding structural information of its source, and we can derive the corresponding similarity levels and frequencies via the extraction procedure. For example, the similarity level of WGPV in terms of protein source *A* is 3 and the frequency is 7. This implies that WGPV has 3 interchangeable amino acids with the corresponding protein word of *A* and it appears 7 times among the similar proteins of *A* found in the PSI-BLAST search result.

**Table 1:** An example of a synonymous word entry in ***SynonymDict.***

Synonymous word: WGPV			
Protein Source	Secondary Structure	Similarity Level	Frequency

*A*	HHHH	3	7
*B*	HHCH	4	11
*C*	CHHH	2	3

An example of a synonymous word entry in *SynonymDict* (assuming the word length *n =* 4). WGPV is a synonymous word of proteins *A, B* and *C*, since it is extracted from the similar proteins of *A, B* and *C.* We record the structural information of protein sources to the corresponding synonymous words, and calculate the corresponding similarity levels and frequencies. For example, the similarity level of WGPV in terms of protein source *A* is 3 and the frequency is 7.

### SymPred: a PSS predictor based on SynonymDict

#### Preprocessing

Given a target protein *t,* whose secondary structure is unknown and to be predicted, we perform a PSI-BLAST search on *t* to compile a word set containing its original protein words and synonymous words. The procedure is similar to the construction of *SynonymDict.* We also calculate the frequency and similarity level of each word in the word set.

#### Exact and inexact matching mechanisms for matching words to *SynonymDict*

Each word *w* in the word set is used to match against words in *SynonymDict,* and the structural information of each protein source in the matched entry is used to vote for the secondary structure of *t.* When matching a word to *SynonymDict,* we consider using straightforward exact matching and a simple inexact matching. Exact matching is rather strict, so we consider a possible relaxation of inexact matching to increase the sensitivity to recover synonymous word matches so that *SynonymDict* can be utilized to more extent than by using exact matching. Our inexact matching allows at most one mismatched character, i.e., allowing a don’t-care character (not a gap) in the words. The matched entries are then evaluated by the following scoring function. (We will compare the two matching mechanisms in Results.)

#### The Scoring Function

To differentiate the effectiveness of matched entries, we design a scoring function based on the protein sources in the matched entries and the sum of the weighted scores on the associated structures determines the predicted structure.

Since we use the structural information of protein sources in the matched entries for structure prediction, we define the scoring function based on its similarity level and frequency recorded in the dictionary for the following observation. *The similarity level represents the degree of similarity between a protein word and its synonymous word, and the frequency represents the degree of sequence conservation in the protein’s evolution.* Intuitively, the greater the similarity between two words, the closer they are in terms of evolution; likewise, the more frequently a word appears in a group of similar proteins, the more conserved it is in terms of evolution.

To define the scoring function, we consider the similarity level and the frequency of the word in the word set of *t,* denoted by *Sim_t_* and *freq_t_* respectively, as well as those of a protein source *i* in its matched entry, denoted by *Sim_i_* and *freq_i_* respectively. Note that *sim_t_* and *freq_t_* are obtained in the preprocessing stage. To measure the effectiveness of the structural information of the protein source *i,* we define the voting score *s_i_* as *min*(*freq_t_, freq_i_*)*×*(1+*min*(*Sim_t_, Sim_i_*_)_)*.* The structural information provided by *i* will be highly effective if: 1) *w* is very similar to the corresponding words of *t* and i; and 2) *w* is well conserved among the similar proteins of *t* and *i.*

Take the synonymous word WGPV in Table 1 as an example. If WGPV is a synonymous word of *t* (assuming *freq_t_* is 5 and *Sim_t_* is 4), then the voting score of the structural information provided by protein source *A* is *min*(5, 7)*×*(1+*min*(4, 3)) = 5×(1+3) = 20. Similarly, the voting score provided by protein source *B* is *min*(5, 11)*×*(1*+min*(4, 4)) = 5×(1+4) = 25, and the score provided by protein source *C* is *min*(5, 3)*×*(1*+min*(4, 2)) = 3×(1+2) = 9. The structural information provided by protein source *B* has the highest score in this matched entry and therefore has the most effect on the prediction.

#### Structure determination

The final structure prediction of the target protein *t* is determined by summing the voting scores of all the protein sources in the matched entries. Specifically, for each amino acid in a protein *t,* we associate three variables, *H*(*x*)*, E*(*x*), and *C*(*x*), which correspond to the total voting scores for the amino acid *x* that has structures H, E, and C, respectively. For example, if we assume that the above synonymous word WGPV is aligned with the residues of protein *t* starting at position 11, then protein *A’s* contribution to the voting score of *H*(11), *H*(12), *H*(13), and *H*(14) would be 20. Similarly, protein *B* would contribute a voting score of 25 to *H*(11), *H*(12), *C*(13), and *H*(14); and protein *C* would contribute a voting score of 9 to *C*(11), *H*(12), *H*(13), and *H*(14). The structure of *x* is predicted to be *H*, *E* or *C* based on *max*(*H*(*x*)*, E*(*x*)*, C*(*x*))*.* When two or more variables have the same highest voting score, C has a higher priority than H, and H has a higher priority than E.

#### Confidence level

A confidence measure of a prediction for each residue is important to a PSS predictor because it reflects the reliability of the predictor’s output. To evaluate the prediction confidence on each amino acid *x*, we calculate a *confidence level* to measure the reliability of the prediction. The confidence level on amino acid x is defined as follows:

The product in the denominator represents a normalization factor for the scoring function. Therefore, the confidence level measures the ratio of the voting scores a residue *x* gets over the summation of the normalization factors. The range of *ConLvl*(*x*) is constrained between 0 and 9 by rounding down. In the Results section, we analyze the correlation coefficient between the confidence level and the average *Q*_3_ accuracy.

### SymPsiPred: a secondary structure meta-predictor

SymPred is different from sequence profile-based methods, such as PSIPRED, which is currently the most popular PSS prediction tool. PSIPRED achieved the top average *Q*_3_ accuracy of 80.6% in the 20 methods evaluated in the CASP4 competition [33]. SymPred and PSIPRED use totally different features and methodologies to predict the secondary structure of a query protein. Specifically, SymPred relies on synonymous words, which represent local similarities among protein sequences and their homologies; however, PSIPRED relies on a position specific scoring matrix (PSSM) generated by PSI-BLAST, which is a condensed representation of a group of aligned sequences. Furthermore, SymPred constructs a protein-dependent synonymous dictionary for inquiries about structural information. In contrast, PSIPRED builds a learning model based on a two-stage neural network to classify sequence profiles into a vector space; thus, it is a probabilistic model of structural types.

It has been shown that combining the prediction results derived by various methods, often referred to as a meta-predictor approach, is a good way to generate better predictions. JPred [34] was the first meta-predictor developed for PSS prediction. After examining the predictions generated by six methods it, JPred returned the consensus prediction result and achieved a 1% improvement over PHD, which was the best single method among the six methods. Similar to the concept of the meta-predictor, we have developed an integrated method called SymPsiPred, which combines the strengths of SymPred and PSIPRED.

To combine the results derived by the two methods, we compare the prediction confidence level of each residue from each method and return the structure with the higher confidence. Since SymPred and PSIPRED use different measures for the confidence levels, we transform their confidence levels into *Q*_3_ accuracies. For each method, we generate an accuracy table showing the average *Q*_3_ accuracy for each confidence level, i.e., we use the average *Q*_3_ accuracy of an SSE to reflect the prediction confidence.

For example, suppose SymPred predicts that a residue in a target sequence has structure *H* with a confidence level of 6, PSIPRED predicts that the residue has structure *E* with a confidence level of 6, and the corresponding *Q*_3_ accuracies in the accuracy tables are 77.6% and 64.6% respectively. In this case, SymPsiPred would predict the residue as *H.*

## Results

In this section, we first reported performance evaluation of SymPred and SymPsiPred on a validation dataset, and then compared our methods with existing methods on EVA benchmark datasets.

### Datasets used to develop SymPred

We downloaded all the protein files in the DSSP database [35] and generated three datasets, i.e., *DsspNr-25, DsspNr-60,* and *DsspNr-90,* based on different levels of sequence identity using the PSI-CD-HIT program [36] following its guidelines. In other words, *DsspNr-25, DsspNr-60* and *DsspNr-90* denote the subset of protein chains in DSSP with mutual sequence identity below 25%, 60% and 90%, respectively, and contain 8297, 12975 and 16391 protein chains, respectively.

### Performance evaluation of SymPred and SymPsiPred on the validation set *DsspNr-25*

We used all the protein chains in *DsspNr-25, DsspNr-60* and *DsspNr-90* as template pools to construct the synonymous dictionaries *SynonymDict-25, SynonymDict-60* and *SynonymDict-90,* respectively. Furthermore, we used *DsspNr-25* as the validation set to determine the parameters of SymPred by leave-one-out cross validation (LOOCV) since LOOCV (also known as *full jack-knife*) has been shown to provide an almost unbiased estimate of the generalization error [37] and makes the most use the data. (SymPred does not need to rebuild model unlike most machine learning methods when using LOOCV.) Once the parameters of SymPred, including the length *n* of a word and the dictionary, were determined, we also used the validation set *DsspNr-25 to* evaluate the performance of SymPred and SymPsiPred by 10-fold cross validation and LOOCV. To avoid over-estimation of SymPred’s performance, when testing each target protein in the *DsspNr-25,* we discarded all the structural information of proteins *t* in the template pool if *t* and the target protein share at least 25% sequence identity.

Choosing the word length 8 with inexact matching criterion and using *SynonymDict-60,* we evaluated the performance of SymPred and SymPsiPred on the validation set *DsspNr-25* by LOOCV and 10-fold cross validation as shown in Table 2. SymPred achieved *Q*_3_ of 80.5% and SOV of 75.6% in 10-fold cross validation and *Q*_3_ of 81.0% and SOV of 76.0% in LOOCV, outperforming PROSP by at least 5.4% in *Q*_3_ and 6.9% in SOV. The meta-predictor, SymPsiPred which integrates the prediction power of SymPred and PSIPRED, achieved a further improvement on *Q*_3_**of 83.9% on *DsspNr-25.* This result demonstrates that SymPsiPred can combines the strengths of the two methods and thus yield much more accurate predictions.

**Table 2:** Performnace comparison of SymPred, SymPsiPred, and PROSP on the *DsspNr-25* dataset.

*DsspNr-25* (8,297 proteins)	*Q*_3_	*Q*_3_Ho	*Q*_3_Eo	*Q*_3_Co	sov	sovH	sovE	sovC
SymPred*	81.0	84.3	71.6	77.7	76.0	82.5	76.9	70.7
SymPred^+^	80.5	84.1	70.9	77.5	75.6	82.3	76.4	70.3
SymPsiPred	83.9	81.5	75.8	83.9	80.2	82.3	80.3	76.5
PROSP	75.1	79.7	67.6	71.3	68.7	77.0	73.0	63.4

Q_3_Ho (Q_3_Eo and Q_3_Co, respectively) represents correctly predicted helix (strand and coil, respectively) residues (percentage of helix observed). sovH/E/C values are the specific SOV accuracies of the predicted helix, strand and coil, respectively. SymPred* represents the experiment result using leave-one-out cross validation and SymPred^+^ represents the experiment result using 10-fold cross validation.

The prediction accuracy of SymPred on *DsspNr-25* was obtained by optimized the two factors: (1) the length of protein words and the matching criterion used for searching the synonymous dictionary and (2) the size of the template pool, as mentioned earlier. Below, we analyze the two factors in more detail and the reported accuracies were obtained by LOOCV.

#### Factor 1: the word length *n* and the matching criterion

The choice of word length *n* is a trade-off between specificity and sensitivity, i.e., long words tend to have highly specific structural features and short words increase sensitivity by recovering sequence matches. Regarding the matching, in the previous study of PROSP, we adopted exact matching when searching a synonymous dictionary. Since the exact matching criterion is rather strict in terms of matching efficiency, we also compared the performance of SymPred using exact matching against using inexact matching, which allows at most one mismatched character.

We evaluated the performance of SymPred using the smallest *SynonymDict-25* dictionary. Table 3 shows the *Q*_3_ accuracy of SymPred with exact and inexact matching on different word lengths. The results reveal that the *Q*_3_ accuracy is not always increasing along the increasing word length in both matching mechanisms. The best *Q*_3_ accuracies are reported at *n*=7 for exact matching and *n*=8 for inexact matching. That is, 7 identical residues yield high specificity for the structural features and a single *don’t-care* character increases the sensitivity to recover sequence matches. In summary, we can improve the prediction performance by using the inexact matching criterion when searching a synonymous dictionary and choosing the word length 8.

**Table 3:** The Q_3_ accuracies of SymPred using exact and inexact matchings on different word lengths.

*Word length n*	*6*	*7*	*8*	*9*
*Q*_3_ (exact matching)	78.2	80.1	78.1	76.2
*Q*_3_ (inexact matching)	74.9	79.2	80.5	79.0

#### Factor 2: the effect of the dataset size used to compile a dictionary

Although the estimated theoretical limit of the accuracy of secondary structure assignment is 88%, current state-of-the-art PSS prediction methods achieve around 80% accuracy; there is an 8% accuracy gap. What is the major obstacle to achieving 88% accuracy? Rost [38] raised this question, and Zhou et al. [39] suggested that the size of an experimental database is crucial to the performance. However, Rost found that PHDpsi trained on only 200 proteins was almost as accurate as PSIPRED trained on 2000 proteins, i.e., the performance is insensitive to the size of the training dataset. This is both a strength and a weakness of machine learning-based approaches. Machine learning-based approaches can generate satisfactory prediction models using a limited dataset. On the other hand, the benefit of using more instances is also limited. Though SymPred is not a machine-learning approach, we still concern the relationship between its performance and the size of a template pool.

We fist studied the sensitivity of the data set size by compiling the *SynonymDict-25* using different percentages of the protein sequences in *DsspNr-25.* (The following analysis is based on word length of 8 and using inexact matching in SymPred.) Table 4 summarizes the prediction performance of SymPred using different percentages of proteins in the template pool. The performance improves as the number of template proteins increases. The *Q*_3_ accuracies for 10% and 100% usage of template proteins are 70.8% and 80.5%, respectively, a 9.7% improvement. Moreover, SymPred’s performance improves between 0.5% and 2.8% each time the number of template proteins is increased by 10%.With more protein sequences in the template pool, the synonymous dictionary can learn more synonymous words from those sequences and their similar protein sequences.

**Table 4:** The Q_3_ accuracy comparison of SymPred using dictionaries compiled from different percentages of the template proteins.

Percentage of template pool	10%	20%	30%	40%	50%	60%	70%	80%	90%	100%
Number of template proteins	830	1660	2490	3320	4150	4980	5809	6638	7467	8297
*Q*_3_ on *DsspNr-25*	70.8	73.6	75.0	76.3	77.3	78.1	78.7	79.3	79.8	80.5
Improvement	-	+2.8	+1.4	+1.3	+1.0	+0.8	+0.6	+0.6	+0.5	+0.7

The performance improves as the number of template proteins increases. SymPred’s performance improves between 0.5% and 2.8% each time the number of template proteins is increased by 10%.

Since SymPred is sensitive to the size of the template pool, we next evaluated its performance on *SynonymDict-60* and *SynonymDict-90,* which were compiled from much larger template pools. Table 5 shows SymPred’s prediction performance using different-sized template pools. Its prediction accuracy reaches 81.0% on *SynonymDict-60,* a 0.5% improvement over using *SynonymDict-25.* We can learn more useful synonymous words from the additional template proteins. The implication is that if protein *A* and protein *B* are similar, say the two share 50% of sequence identity, then PSI-BLAST can find more similar protein sequences by analyzing *A* and *B* together, rather than separately. For example, there might be a protein *C* that is only similar to protein *B.* In such a case, if *A* is the query sequence, PSI-BLAST would not report protein *C* due to the low sequence identity. However, the advantage decreases when a larger number of similar proteins are involved in the template pool, as shown by the result for *SynonymDict-90,* which is comprised of proteins whose sequence identities are below 90%. The sequence conservation rate contracts to highly similar sequences, and this leads to a bias in the weighted scores of the scoring system. Therefore, we adopt *SynonymDict-60* as the primary synonymous dictionary for making predictions.

**Table 5:** Comparison of SymPred’s prediction performance on different-sized template pools.

*Template pool*	*DsspNr-25*	*DsspNr-60*	*DsspNr-90*
Number of template proteins	8297	12975	16391
Synonymous dictionary	*SynonymDict-25*	*SynonymDict-60*	*SynonymDict-90*

**Q_3_ on *DsspNr-25***	**80.5**	**81.0**	**80.9**

### Evaluation of the confidence level

**Figure 3:**
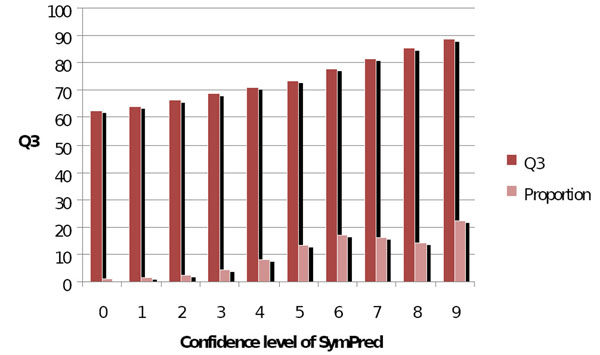
**Relationships between *Q*_3_ accuracy and confidence level on SymPred**. The correlation coefficient between the confidence levels and *Q*_3_ accuracies for SymPred is 0.992.

Figure 3 shows the utility of our confidence level in judging the prediction accuracy of each residue in the validation set. The statistics are based on more than 2 million residues. The correlation coefficient between the confidence levels and *Q*_3_ accuracies for SymPred is 0.992. Thus, our method provides strong confidence measures for the output. We observe that a confidence level of 7 or above reported by SymPred is attributed to 53% of the residues with more than 81% of the *Q*_3_ accuracy.

### Performance comparison with existing methods on EVA benchmark datasets

EVA test sets usually serve as benchmarks of protein secondary structure predictors, particular for CASP competitions [40]. Only proteins without significant sequence identity to previously known PDB proteins were used to test on different existing methods. We chose two latest EVA sequence-unique subsets of the PDB, called *EVA _Set1* (protein list: http://cubic.bioc.columbia.edu/eva/sec/set_com1.html) and *EVA_Set2* (protein list: http://cubic.bioc.columbia.edu/eva/sec/set_com6.html), the former containing 80 proteins tested on the most number of methods and the latter with the maximum number of proteins (212 proteins). The two datasets serve as independent test sets for performance comparison of SymPred with other existing methods.

#### Benchmark comparison results

For fair comparison, when predicting the secondary structure of each target protein in an independent set, SymPred discarded the structural information of all proteins sharing at least 25% of the sequence identity with the target protein in the template pool, i.e., SymPred used in the template pool the structural information of proteins sharing no more than 25% sequence identity with the target protein.

Table 6 shows the experiment result on the two benchmark datasets, *EVA_ Set 1* and *EVA_Set2,* where SymPred’s results were achieved by using *n=* 8, inexact matching and *SynonymDict-60* It shows that SymPred achieves *Q*_3_ accuracies of 78.8% (SOV=76.4%) and 79.2% (SOV=76.0%), outperforming existing state-of-the-art methods by 1.4% to 5.4%. It can be observed that SymPred performs better than each single predictor on most of performance measurements.

**Table 6:** The prediction performance of different methods on the EVA benchmark datasets.

*EVA_Set1* (80 proteins)	*Q*_3_	*ERRsig Q*_3_	sov	*ERRsig* sov	sovH	sovE	sovC
SymPred	78.8	±1.4	76.4	±1.9	85.0	76.5	70.4
SAM-T99sec	77.2	±1.2	74.6	±1.5	80.9	72.5	71.2
PSIPRED	76.8	±1.4	75.4	±2.0	82.1	72.3	65.2
PROFsec	75.5	±1.4	74.9	±1.9	78.3	75.9	71.3
PHDpsi	73.4	±1.4	69.5	±1.9	73.7	73.9	65.2

							

*EVA_Set2* (212 proteins)	*Q*_3_	*ERRsig Q*_3_	sov	*ERRsig* sov	sovH	sovE	sovC

SymPred	79.2	±0.9	76.0	±1.2	85.1	77.7	71.3
PSIPRED	77.8	±0.8	75.4	±1.1	80.6	72.6	70.4
PROFsec	76.7	±0.8	74.8	±1.1	79.2	76.2	71.8
PHDpsi	75.0	±0.8	70.9	±1.2	77.0	72.4	67.0

sovH/E/C values are the specific SOV accuracies of the predicted helix, strand and coil, respectively. The prediction results of other methods on *EVA_Set 1* and *EVA_Set2* are reported at http://cubic.bioc.columbia.edu/eva/sec/common3.html.

## Discussions

In this section, we analyze the prediction power of SymPred on similar proteins as well as the relationship between the number of synonymous words and the method’s prediction performance. We also demonstrate the structure conservation of synonymous words via a case study of a pair of protein sequences that are very dissimilar at the sequence level.

### Evaluation on similar proteins

One weakness of machine learning-based methods is that they may under-utilize the structural information in the training set when the query protein has a high sequence similarity to a template in the training set. Therefore, we assess the performance of SymPred when there are sequence similarities between test proteins and proteins in the template pool. Since *SynonymDict-90* contains the largest number of known-structure protein sequences, we conducted an experiment in which we used all the structural information of the template proteins in the dictionary, except the information of the target protein itself. Of the 8297 target proteins, 3585 have similar proteins in the template pool (i.e., the sequence identity ≥25%). SymPred’s average *Q*_3_ accuracy on those proteins is 88.1%, which fits the estimated theoretical limit of the accuracy. The result shows that SymPred can utilize the structural information in the template pool effectively when there are sequence similarities to the target protein sequence.

### Prediction accuracy affected by enlargement of synonymous words

Although the parameter *b* in PSI-BLAST is set at 500 for searches, not every query protein can have that number of similar proteins in the database used to generate sequence alignments. Because some query proteins are quite unique, PSI-BLAST only reports a few similar proteins at most, and may not report any. In such cases, SymPred would not have enough synonymous words to generate reliable predictions. On the other hand, some query proteins have many highly similar proteins in the database, which results in duplicate synonymous words. Apart from the number of sequence alignments, the number of distinct synonymous words may affect SymPred’s performance. Therefore, we analyze the relationship between the number of distinct synonymous words and the SymPred’s prediction performance.

To study the relationship, we set different thresholds for selecting corresponding subsets *u* of test protein sequences. The selection criterion is defined as follows. For each test protein *t* in *DsspNr-25,* let v denote the number of distinct synonymous words in the word set of *t,* and let *L* be the sequence length of *t*; then let *e = v/L,* which denotes the multiple of *L* in terms of *v*. If *e* is greater than or equal to a threshold, the protein *t* is added to *u.* We compare the average *Q*_3_ accuracy of proteins in *u* with respect to different thresholds.

Table 7 shows the prediction performance of SymPred and SymPsiPred with respect to different thresholds. The results show that there is a positive correlation between the number of distinct synonymous words and the prediction performance of SymPred and SymPsiPred. For SymPred, the accuracy improves from 81.0% to 83.5% when the threshold increases from e≥0 to e≥150. It is remarkable that SymPred can predict approximately 75% of the proteins in *DsspNr-25* with 83.1% accuracy, and more than 50% of the protein sequences can be predicted with 83.5% accuracy. For SymPsiPred, the accuracy increases from 83.9% to 85.5% when the threshold increases from e ≥ 0 to *e* ≥ 150. The results imply that SymPred and SymPsiPred have the potential to achieve higher accuracy as the number of protein sequences in the NCBInr database increases.

**Table 7:** The relationship between the number of distinct synonymous words and the prediction performance.

Selection criterion		***e*** **≥0**	*e*≥5	*e*≥25	*e*≥50	*e*≥75	*e*≥100	*e*≥125	*e*≥150
Number of selected proteins		8297	7983	7252	6660	6178	5637	5035	4378

*Q*_3_	SymPred	81.0	81.6	82.3	82.8	83.1	83.3	83.4	83.5

	SymPsiPred	83.9	84.3	84.8	85.1	85.2	85.3	85.4	85.5

For each test protein *t* of length *L* in *DsspNr-25*, let v denote the number of distinct synonymous words of *t.* Define *e = v/L,* the multiplicity of *v* over *L.* If *e* is greater than or equal to a threshold, the protein *t* is selected. The results show that there is a positive correlation between the number of distinct synonymous words and the prediction performance of SymPred and SymPsiPred.

### Sequence alignment by using synonymous words

From the performance of SymPred, we observe that protein-dependent synonymous words possess the property of structure conservation. In other words, the synonymous words show the semantic relationship in terms of protein structures. To further demonstrate the structure conservation property, we compare the synonymous words of two proteins and analyze the shared synonymous words with respect to each residue pair of the two proteins. The distribution of shared synonymous words can help to generate a highly accurate alignment for two protein sequences.

Balibase 3.0 [41], a database that serves as an evaluation resource for sequence alignments, contains manually constructed multiple sequence alignments that are all based on three-dimensional structural superpositions. Therefore, Balibase can be used as a benchmark of sequence alignment tools. We downloaded the first test case (BB11001) and used the first two proteins (1aab and 1j46_A) to demonstrate the structure conservation of synonymous words. The sequence identity of the two proteins is only 16.7%; however, they belong to the same Family (HMG-box) according to the SCOP classification. This indicates that the two proteins are remotely homologous.

**Figure 4:**
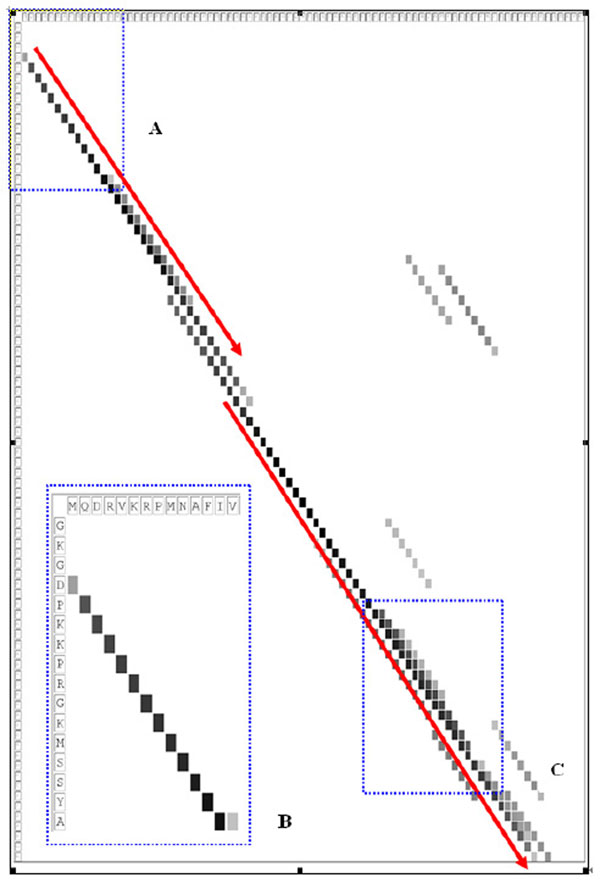
**The distribution of synonymous words shared by 1aab and 1j46_A.** The x- and y- axes represent the sequence of 1j46_A and 1aab respectively. A grayscale pixel represents the number of shared synonymous words corresponding to a residue pair (*x_i_, y_j_*), where *x*_i_ and *y_j_* denote a residue pair comprised of the *i*-th residue of 1j46_A and the *j*-th residue of 1aab respectively. Box B is a zoom-in of Box A. The red lines indicate the alignment based on the number of shared synonymous words, and the alignment is very close to that reported in Balibase for the two proteins. Notably, it can be observed that the path of the darker pixels is nearly perfectly matched the suggested alignment.

Figure 4 shows the distribution of synonymous words shared by the two proteins. The x- and y- axes represent the sequence of 1j46_A and 1aab respectively. A grayscale pixel represents the number of shared synonymous words corresponding to a residue pair (*x_i_*, *y_j_*), where *x_i_* and *y_j_* denote a residue pair comprised of the *i*-th residue of 1j46_A and the *j*-th residue of 1aab respectively. More specifically, if an identical synonymous word *sw* of length *w* is both derived from 1j46_A and 1aab beginning with residue *x_i_* and *y_j_* respectively, then the residue pairs (*x_i_*, *y_j_*), (*x_i_*_+1_, *y_j_*_+1_), …, and (*x_i_*_+_*_w_*_-1_, *y_j_*_+_*_w_*_-1_) are all counted to share *sw.* The darker the pixel, the greater the number synonymous words shared by *x_i_* and *y_j_.*

In the figure, Box B is a zoom-in of Box A. We can see that the fourth residue of 1j46_A shares some synonymous words with the first residue of 1aab, the fifth residue of 1j46_A shares more synonymous words with the second residue of 1aab, and so on. It is noteworthy that the Box C shows some residues of 1j46_A shares synonymous words with multiple and continuous residues of 1aab. Since the experiment results suggest that synonymous words are likely expressing similar structures, the Box C implies a possible tolerance of deletions in protein 1aab.

We align the two sequences based on the distribution of synonymous words shared by the two sequences. Instead of using a substitution matrix to calculate the score of an aligned residue pair, we use the number of shared synonymous words between a residue pair since the number of shared synonymous words can reflect both the sequence and the structure similarities of a residue pair. As a result, it generates an alignment indicated by the red lines shown in the figure, i.e., the fourth residue of 1j46_A is aligned with the first residue of 1aab, the fifth residue of 1j46_A with the second residue of 1aab, etc, and there are two gaps in the midst of the alignment. (The red lines are drawn shifted a little bit in order to avoid overlapping the dark pixels.) Notably, the resulting alignment is very close to the alignment reported in Balibase for the two proteins, matching 76 out of 78 correct residues pairs, i.e., 97% of alignment accuracy, while ClustalW aligns 64 out of 78 residue pairs (82.1% accuracy) correctly. More examples of highly accurate alignment by using synonymous words could be found in other protein pairs. Overall speaking, the distribution of shared synonymous words could indicate three-dimensional structural superpositions as well as the possible alignment of a protein sequence pair.

## Conclusions

In this paper, we have proposed an improved dictionary-based approach called SymPred for PSS prediction. We have also presented a meta-predictor called SymPsiPred, which combines a dictionary-based approach (SymPred) and a machine learning-based approach (PSIPRED). Tests on a proteome-scale dataset of 8297 protein chains show that the overall average *Q*_3_ accuracy of SymPred and SymPsiPred is 81.0% and 83.9% respectively. Through the blind test on the two independent test sets, SymPred achieves the average *Q*_3_ accuracies of 78.8% and 79.2% respectively, which are better than other state-of-the-art PSS predictors. SymPred can be regarded as a special case of a template-based approach because it predicts PSS by finding template sequences based on local similarities, i.e., synonymous words. However, the accuracy gap between the template-based methods and machine learning-based methods is approximately 10%. We show that SymPred can reduce that gap by using n-gram patterns.

From the analysis of two factors, we find that the prediction accuracy of SymPred can be gradually improved based on each factor’s optimization. In particular, SymPred is very sensitive to the size of the template pool, as shown by the fact that its performance improves between 0.5% and 2.8% each time the number of template proteins is increased by 10%. Therefore, the performance accuracy will improve further as the number of known-structure proteins increases. Furthermore, from the analysis of the number of distinct synonymous words, we posit that, as the number of protein sequences of unknown structures increases in the NCBInr database, we will be able to discover more sequence variations and derive more synonymous words to improve SymPred’s performance. The average *Q*_3_ accuracy of SymPred is above 83% for proteins that have synonymous words satisfying *e* ≥ 75. Meanwhile, the *Q*_3_ accuracy of SymPsiPred is above 85%, which is even closer to the estimated theoretical limit of PSS prediction accuracy. The results imply that SymPred and SymPsiPred have the potential to achieve higher accuracy as the number of protein sequences in the PDB database and the NCBInr database increases.

When SymPred is tested on proteins that have sequence similarities to the template proteins, the average *Q*_3_ accuracy is approximately 88%. The result shows that SymPred can utilize the structural information in the template pool effectively. We also demonstrate the power of synonymous words in the sequence comparisons. The information about shared synonymous words can be used to infer three-dimensional structural superpositions. The experiments and the analysis results indicate that synonymous words are reliable short templates that can provide protein-related information.

A major advantage of dictionary-based methods is that the prediction process is transparent and easy to understand. Unlike machine learning-based methods, which are computationally intractable, we can examine the prediction process to observe how SymPred generates predictions, including the synonymous words it matches against the dictionary and the template proteins involved in the prediction process. To differentiate the prediction model from machine learning-based methods, it is often referred to as a black box model. Another major advantage of dictionary-based methods is that adding more proteins with known structures is much easier than under machine learning-based methods. Unlike most machine learning-based methods, which need to retrain the prediction models, the proposed dictionary-based method can be expanded incrementally by simply adding new synonymous words or by updating existing entries with new protein sources and the associated structural information.

## Competing interests

The authors declare that they have no competing interests.

## Authors' contributions

Hsin-Nan Lin developed the method, carried out the computational predictions. Hsin-Nan Lin and Ting-Yi Sung were involved in the literature survey, result interpretation, and manuscript writing. Ting-Yi Sung, Shinn-Ying Ho and Wen-Lian Hsu coordinated the study and revised the manuscript. All authors read and approved the final manuscript.
